# Supplementary material for: Analysis of PPARγ Signaling Activity in Psoriasis
Source: Int J Mol Sci. 2021 Aug 10;22(16):8603. doi: 10.3390/ijms22168603 (PMC8395241; doi:10.3390/ijms22168603)
Supplement: Supplementary file 1 [file ijms-22-08603-s001.zip › Supplemental materials_Analysis of PPARg signaling activity in psoriasis/Pathway models/Models images and html files/Model of PPARG signaling in psoriasis/13361.html]

TNFSF10


# Protein TNFSF10

|  |  |
| --- | --- |
| URN | urn:agi-llid:8743 |
| Total Entities | 0 |
| Connectivity | 4117 |
| Name | TNFSF10 |
| Description | tumor necrosis factor (ligand) superfamily, member 10 |
| Notes | The protein encoded by this gene is a cytokine that belongs to the tumor necrosis factor (TNF) ligand family. This protein preferentially induces apoptosis in transformed and tumor cells, but does not appear to kill normal cells although it is expressed at a significant level in most normal tissues. This protein binds to several members of TNF receptor superfamily including TNFRSF10A/TRAILR1, TNFRSF10B/TRAILR2, TNFRSF10C/TRAILR3, TNFRSF10D/TRAILR4, and possibly also to TNFRSF11B/OPG. The activity of this protein may be modulated by binding to the decoy receptors TNFRSF10C/TRAILR3, TNFRSF10D/TRAILR4, and TNFRSF11B/OPG that cannot induce apoptosis. The binding of this protein to its receptors has been shown to trigger the activation of MAPK8/JNK, caspase 8, and caspase 3. Alternatively spliced transcript variants encoding different isoforms have been found for this gene. [provided by RefSeq, Jul 2010] |
| Primary Cell Localization | Extracellular |
| Class | Ligand |
| ObjectType | Protein |

---

|  |  |
| --- | --- |
| Pathway | Cortisol in Resolving Inflammation |
|  | Toll-like Receptors Act through MYD88-TIRAP Signaling |
|  | TLR4 Signaling in Leukocytes |
|  | Natural Killer Cell Activation |
|  | Peripheral T-Cell Tolerance |
|  | T-Cell Central Tolerance |
|  | T-Cell Cytotoxic Mediated Cell Death |
|  | Toll-like Receptors in Sterile Inflammation |
|  | Megakaryocyte -> Platelet Precursor Cell Surface Expression Markers |
|  | T-lymphoid precursor cell -> CD8+ Naive T-cell Surface Expression Markers |
|  | Myoblast -> Neutrophil Surface Expression Markers |
|  | Apoptosis |
|  | Proteins Involved in Atherosclerosis |
|  | Apoptosis of Myocyte |
|  | Proteins Involved in Melanoma |
|  | Dendritic Cells Function in Psoriasis |
|  | Proteins Involved in Systemic Lupus Erythematosus |
|  | Proteins Involved in Diabetes Mellitus Type 1 |
|  | Proteins Involved in Diabetic Nephropathy |
|  | Proteins Involved in Colorectal Neoplasms |
|  | Crohn's Disease |
|  | Dendritic Cell Dysfunction in Crohn's disease |
|  | Proteins Involved in Multiple Myeloma |
|  | Proteins Involved in Pancreatic Cancer |
|  | Apoptosis Block in Synovial Fibroblast |
|  | Proteins Involved in Rheumatoid Arthritis |
|  | Proteins Involved in HIV Type 1 Infection |
|  | Neurofibrillary Tangle Formation |
|  | Proteins Involved in Glioblastoma |
|  | Proteins Involved in Astrocytoma |
|  | Proteins Involved in Glioma |
|  | Proteins Involved in Medulloblastoma |
|  | Proteins Involved in Neuroblastoma |
|  | Proteins Involved in Multiple Sclerosis |
|  | Breast Cancer |
|  | Proteins Involved in Prostate Cancer |
|  | CXCL12 Expression Targets |
|  | Clozapine Induced Granulocytopenia |
|  | EGF -> FOXO3A Expression Targets |
|  | Erythropoietin -> FOXO3A Expression Targets |
|  | F2 -> AP-1/CREB/ELK/SRF/SP1 Expression Targets |
|  | F2 -> STAT1/NF-kB Expression Targets |
|  | Apoptosis Block by Death Receptor Signaling in Cancer |
|  | Apoptosis Evasion in Cancer: Overview |
|  | Treg Cells Promote Immunosuppression in Cancer Immune Escape |
|  | Insulin -> CEBPA/CTNNB/FOXA/FOXO Expression Targets |
|  | IFNA1/IFNR Expression Targets |
|  | IFNG/IFNR Expression Targets |
|  | IL13 Expression Targets |
|  | IL15 Expression Targets |
|  | IL2 Expression Targets |
|  | Morphine Expression Targets |
|  | IFNA1/Gq Expression Targets |
|  | TCR -> CREB/CREBBP/ATF Expression Targets |
|  | TCR -> NF-kB Expression Targets |
|  | TCR -> STAT Expression Targets |
|  | TCR -> AP-1 Expression Targets |
|  | TGFB1-TGFBR2 Expression Targets |
|  | TGFB1-TGFBR1/AP-1 Expression Targets |
|  | TGFB1-ACVRL1 Expression Targets |
|  | TNFSF10 Expression Targets |
|  | TNF -> AP-1 Expression Targets |
|  | TNFR -> CREB/ELK-SRF Signaling |
|  | TNFR -> AP-1/ATF/TP53 Signaling |
|  | TNFR -> NF-kB Signaling |
|  | TLR1 -> 2/6 Expression Targets |
|  | TLR4 -> NF-kB/IRF Expression Targets |
|  | prarg negative regulators, ps-positive |
|  | PPARG negative regulators |
|  | pparg expr target\_regulators |
|  | Neighbors of disease exacerbation |
|  | Neighbors of tumor progression |
|  | Neighbors of cancer progression |
|  | Neighbors of inflammatory status |
|  | Neighbors of overall survival |
|  | Neighbors of disease severity |
|  | Neighbors of tumor size |
|  | Neighbors of lesion size |
|  | Neighbors of mortality |
|  | Neighbors of survival rate |
|  | Neighbors of survival time |
|  | Neighbors of functional recovery |
|  | Neighbors of insulin sensitivity |
|  | Neighbors of cancer survival |
|  | Neighbors of graft survival |
|  | Neighbors of therapeutic efficacy |
|  | Neighbors of lethality |
|  | Neighbors of tumor regression |
|  | Neighbors of recurrence-free survival |
|  | Neighbors of disease-free survival |
|  | Neighbors of tumor response |
|  | Neighbors of long-term survival |
|  | Neighbors of treatment outcome |
|  | Neighbors of body weight |
|  | Neighbors of body weight gain |
|  | Neighbors of cardiovascular risk |
|  | Neighbors of microvessel density |
|  | Neighbors of neutrophil count |
|  | Neighbors of morbidity |
|  | Neighbors of mortality rate |
|  | Neighbors of fat mass |
|  | Neighbors of bone mineral density |
|  | Neighbors of cell density |
|  | Neighbors of progression-free survival |
|  | Neighbors of apoptosis rate |
|  | Neighbors of early mortality |
|  | Neighbors of viral load |
|  | Neighbors of blood pressure |
|  | Neighbors of vascular reactivity |
|  | Neighbors of cancer incidence |
|  | Neighbors of systolic blood pressure |
|  | Neighbors of tumor resistance |
|  | Neighbors of blood glucose |
|  | Neighbors of median survival |
|  | Neighbors of fasting plasma glucose |
|  | Neighbors of osteoclast number |
|  | Neighbors of antimetastatic activity |
|  | Neighbors of glucose level |
|  | Neighbors of radiosensitivity |
|  | Neighbors of pregnancy outcome |
|  | Neighbors of fertility |
|  | Neighbors of monocyte count |
|  | Neighbors of metabolic response |
|  | Neighbors of disease exacerbation |
|  | Neighbors of tumor progression |
|  | Neighbors of cancer progression |
|  | Neighbors of inflammatory status |
|  | Neighbors of overall survival |
|  | Neighbors of disease severity |
|  | Neighbors of tumor size |
|  | Neighbors of lesion size |
|  | Neighbors of mortality |
|  | Neighbors of survival rate |
|  | Neighbors of survival time |
|  | Neighbors of functional recovery |
|  | Neighbors of insulin sensitivity |
|  | Neighbors of cancer survival |
|  | Neighbors of graft survival |
|  | Neighbors of therapeutic efficacy |
|  | Neighbors of lethality |
|  | Neighbors of tumor regression |
|  | Neighbors of recurrence-free survival |
|  | Neighbors of disease-free survival |
|  | Neighbors of tumor response |
|  | Neighbors of long-term survival |
|  | Neighbors of treatment outcome |
|  | Neighbors of body weight |
|  | Neighbors of body weight gain |
|  | Neighbors of cardiovascular risk |
|  | Neighbors of microvessel density |
|  | Neighbors of neutrophil count |
|  | Neighbors of morbidity |
|  | Neighbors of mortality rate |
|  | Neighbors of fat mass |
|  | Neighbors of bone mineral density |
|  | Neighbors of cell density |
|  | Neighbors of progression-free survival |
|  | Neighbors of apoptosis rate |
|  | Neighbors of early mortality |
|  | Neighbors of viral load |
|  | Neighbors of blood pressure |
|  | Neighbors of vascular reactivity |
|  | Neighbors of cancer incidence |
|  | Neighbors of systolic blood pressure |
|  | Neighbors of tumor resistance |
|  | Neighbors of blood glucose |
|  | Neighbors of median survival |
|  | Neighbors of fasting plasma glucose |
|  | Neighbors of osteoclast number |
|  | Neighbors of antimetastatic activity |
|  | Neighbors of glucose level |
|  | Neighbors of radiosensitivity |
|  | Neighbors of pregnancy outcome |
|  | Neighbors of fertility |
|  | Neighbors of monocyte count |
|  | Neighbors of metabolic response |
|  | Neighbors of cell development |
|  | Neighbors of immune response |
|  | Neighbors of cell population |
|  | Neighbors of T-cell development |
|  | Neighbors of inflammatory response |
|  | Neighbors of angiogenesis |
|  | Neighbors of innate immune response |
|  | Neighbors of cell function |
|  | Neighbors of macrophage activation |
|  | Neighbors of cell formation |
|  | Neighbors of adaptive immune response |
|  | Neighbors of osteoclast development |
|  | Neighbors of epithelial to mesenchymal transition |
|  | Neighbors of immunity |
|  | Neighbors of cell proliferative response |
|  | Neighbors of chemotaxis |
|  | Neighbors of dendritic cell differentiation |
|  | Neighbors of cell survival |
|  | Neighbors of osteoclast differentiation |
|  | Neighbors of cell phenotype |
|  | Neighbors of T-cell proliferation |
|  | Neighbors of tissue remodeling |
|  | Neighbors of cell adhesion |
|  | Neighbors of T-cell activation |
|  | Neighbors of endothelial cell proliferation |
|  | Neighbors of fibrogenesis |
|  | Neighbors of tumor growth |
|  | Neighbors of dendritic cell development |
|  | Neighbors of tissue repair |
|  | Neighbors of cell count |
|  | Neighbors of cell infiltration |
|  | Neighbors of cell interaction |
|  | Neighbors of SMC proliferation |
|  | Neighbors of lumen formation |
|  | Neighbors of endothelial cell migration |
|  | Neighbors of adipogenesis |
|  | Neighbors of lipid storage |
|  | Neighbors of cell damage |
|  | Neighbors of pregnancy |
|  | Neighbors of aging |
|  | Neighbors of cell motility |
|  | Neighbors of bone resorption |
|  | Neighbors of monocyte migration |
|  | Neighbors of adipocyte differentiation |
|  | Neighbors of macrophage migration |
|  | Neighbors of microglial activation |
|  | Neighbors of regeneration |
|  | Neighbors of cell migration |
|  | Neighbors of cancer growth |
|  | Neighbors of smooth muscle cell migration |
|  | Neighbors of hepatic regeneration |
|  | Neighbors of transendothelial migration |
|  | Neighbors of phagocytosis |
|  | Neighbors of immune system activation |
|  | Neighbors of leukocyte migration |
|  | Neighbors of leukocyte recruitment |
|  | Neighbors of osteoclast formation |
|  | Neighbors of monocyte recruitment |
|  | Neighbors of cell invasion |
|  | Neighbors of hemopoiesis |
|  | Neighbors of bone remodeling |
|  | Neighbors of T-cell function |
|  | Neighbors of immune system function |
|  | Neighbors of autophagy |
|  | Neighbors of monocyte adhesion |
|  | Neighbors of transcription activation |
|  | Neighbors of senescence |
|  | Neighbors of T-cell response |
|  | Neighbors of tumor immunity |
|  | Neighbors of cell transdifferentiation |
|  | Neighbors of neuroprotection |
|  | Neighbors of endothelial cell function |
|  | Neighbors of neuronal death |
|  | Neighbors of cell growth |
|  | Neighbors of ROS generation |
|  | Neighbors of first trimester pregnancy |
|  | Neighbors of cancer cell growth |
|  | Neighbors of sensitization |
|  | Neighbors of hemato-encephalic barrier |
|  | Neighbors of macrophage function |
|  | Neighbors of cellular immune response |
|  | Neighbors of cellular senescence |
|  | Neighbors of colony formation |
|  | Neighbors of cell differentiation |
|  | Neighbors of keratinocyte proliferation |
|  | Neighbors of stem cell migration |
|  | Neighbors of life span |
|  | Neighbors of cell homeostasis |
|  | Neighbors of psoriasis |
|  | Neighbors of inflammatory disease |
|  | Neighbors of rheumatoid arthritis |
|  | Neighbors of atherosclerosis |
|  | Neighbors of fibrosis |
|  | Neighbors of colitis |
|  | Neighbors of arthritis |
|  | Neighbors of inflammatory bowel disease |
|  | Neighbors of injury |
|  | Neighbors of atherogenesis |
|  | Neighbors of vascular remodeling |
|  | Neighbors of metastasis |
|  | Neighbors of carcinogenesis |
|  | Neighbors of multiple sclerosis |
|  | Neighbors of chronic inflammation |
|  | Neighbors of inflammation |
|  | Neighbors of tumor microenvironment |
|  | Neighbors of pathological angiogenesis |
|  | Neighbors of obesity |
|  | Neighbors of vascular disease |
|  | Neighbors of diabetes mellitus |
|  | Neighbors of asthma |
|  | Neighbors of pneumonia |
|  | Neighbors of hyperplasia |
|  | Neighbors of osteoarthritis |
|  | Neighbors of liver fibrosis |
|  | Neighbors of chronic obstructive pulmonary disease |
|  | Neighbors of leukocyte infiltration |
|  | Neighbors of reperfusion injury |
|  | Neighbors of airway inflammation |
|  | Neighbors of hepatitis |
|  | Neighbors of insulin resistance |
|  | Neighbors of infection |
|  | Neighbors of vascular injury |
|  | Neighbors of renal injury |
|  | Neighbors of preeclampsia |
|  | Neighbors of macrophage infiltration |
|  | Neighbors of liver injury |
|  | Neighbors of neuroinflammation |
|  | Neighbors of sepsis |
|  | Neighbors of death |
|  | Neighbors of type 1 diabetes |
|  | Neighbors of encephalomyelitis |
|  | Neighbors of autoimmune disease |
|  | Neighbors of ischemic stroke |
|  | Neighbors of neutrophil infiltration |
|  | Neighbors of diabetic nephropathy |
|  | Neighbors of lung injury |
|  | Neighbors of immunopathology |
|  | Neighbors of systemic sclerosis |
|  | Neighbors of hepatocellular carcinoma |
|  | Neighbors of autoimmunity |
|  | Neighbors of acute lung injury |
|  | Neighbors of gastric cancer |
|  | Neighbors of atherosclerotic plaque |
|  | Neighbors of experimental autoimmune encephalomyelitis |
|  | Neighbors of colorectal cancer |
|  | Neighbors of breast cancer |
|  | Neighbors of ischemia |
|  | Neighbors of systemic lupus erythematosus |
|  | Neighbors of melanoma |
|  | Neighbors of collagen-induced arthritis |
|  | Neighbors of neoplasm invasion |
|  | Neighbors of kidney disease |
|  | Neighbors of pulmonary fibrosis |
|  | Neighbors of cardiovascular disease |
|  | Neighbors of stroke |
|  | Neighbors of cancer |
|  | Neighbors of type 2 diabetes |
|  | Neighbors of neoplasm |
|  | Neighbors of neointima |
|  | Neighbors of vasculitis |
|  | Neighbors of respiratory hypersensitivity |
|  | Neighbors of myocardial fibrosis |
|  | Neighbors of coronary artery disease |
|  | Neighbors of endothelial cell dysfunction |
|  | Neighbors of liver disease |
|  | Neighbors of prostate cancer |
|  | Neighbors of hypertrophy |
|  | Neighbors of osteolysis |
|  | Neighbors of ovarian cancer |
|  | Neighbors of pancreatic cancer |
|  | Neighbors of hypertension |
|  | Neighbors of allograft rejection |
|  | Neighbors of epithelial cell |
|  | Neighbors of keratinocyte |
|  | Neighbors of vascular smooth muscle cell |
|  | Neighbors of umbilical vein endothelial cell |
|  | Neighbors of monocyte |
|  | Neighbors of alveolar macrophage |
|  | Neighbors of macrophage |
|  | Neighbors of fibroblast |
|  | Neighbors of airway epithelial cell |
|  | Neighbors of PBMC |
|  | Neighbors of airway smooth muscle cell |
|  | Neighbors of osteoblast |
|  | Neighbors of microglia |
|  | Neighbors of trophoblast |
|  | Neighbors of hepatic stellate cell |
|  | Neighbors of hepatocyte |
|  | Neighbors of endothelial cell |
|  | Neighbors of dendritic cell |
|  | Neighbors of kidney tubule cell |
|  | Neighbors of inflammatory cell |
|  | Neighbors of osteoclast |
|  | Neighbors of neutrophil |
|  | Neighbors of smooth muscle myocyte |
|  | Neighbors of astrocyte |
|  | Neighbors of activated T-cell |
|  | Neighbors of immunocompetent cell |
|  | Neighbors of granulocyte |
|  | Neighbors of myeloid cell |
|  | Neighbors of mesenchymal stroma cell |
|  | Neighbors of cytotrophoblast |
|  | Neighbors of stem cell |
|  | Neighbors of leukocyte |
|  | Neighbors of T-cell |
|  | Neighbors of pancreatic stellate cell |
|  | Neighbors of mast cell |
|  | Neighbors of podocyte |
|  | Neighbors of antigen-presenting cell |
|  | Neighbors of phagocyte |
|  | Neighbors of lymphocyte |
|  | Neighbors of lymphoid cell |
|  | Neighbors of lung |
|  | Neighbors of liver |
|  | Neighbors of carotid artery |
|  | Neighbors of blood vessel wall |
|  | Neighbors of islets of Langerhans |
|  | Neighbors of lymphatic system |
|  | Neighbors of spleen |
|  | Neighbors of alveolus |
|  | Neighbors of hip region |
|  | Neighbors of NF-kB family |
|  | Neighbors of oxidized LDL |
|  | Neighbors of JNK |
|  | Neighbors of IL1 family |
|  | Neighbors of PPAR |
|  | Neighbors of Jun/Fos |
|  | Neighbors of PKC |
|  | Neighbors of PI3K |
|  | Neighbors of STAT family |
|  | Neighbors of IFNAR ligand |
|  | Neighbors of transforming growth factor |
|  | Neighbors of cytokine |
|  | Neighbors of interferon |
|  | Neighbors of interleukin |
|  | Neighbors of growth factor |
|  | Neighbors of NF-AT family |
|  | Neighbors of beta adrenoceptor |
|  | Neighbors of adipokine |
|  | Neighbors of protein tyrosine kinase |
|  | Neighbors of IgG |
|  | Neighbors of SMAD subfamily |
|  | Neighbors of inflammatory cytokine |
|  | Neighbors of estrogen receptor |
|  | Neighbors of T-cell receptor |
|  | Neighbors of IL12 |
|  | Neighbors of lectin |
|  | Neighbors of interleukin-35 |
|  | Neighbors of proteasome endopeptidase complex |
|  | Neighbors of histone H3 |
|  | Neighbors of WNT |
|  | Neighbors of PP2B |
|  | Neighbors of ITG |
|  | Neighbors of CD3 complex |
|  | Neighbors of histone |
|  | Neighbors of deacetylase |
|  | Model of PPARG signaling in psoriasis |
|  | PPARG negative regulators and targets |
|  | dsRNA induces innate immunity and INFs in alveolar cells |
|  | 4\_2\_Genes with mutations currently associated with ulcerative colitis |
|  | 4\_1\_Polymorphisms associated with inflammatory bowel diseases |
|  | 2\_Lipotoxicity in NAFLD |
|  | 2\_HIV - induced depletion of CD4+ T-cells |
|  | 1\_Antigen-presenting cells promote islet dysfunction and immune system activation in T1D |
|  | drug-target moltransport |

---

|  |  |
| --- | --- |
| MedScan ID | 8743 |

---

|  |  |
| --- | --- |
| LocusLink ID | 8743 |
|  | 22035 |
|  | 246775 |

---

|  |  |
| --- | --- |
| Alias | tumor necrosis factor-related apoptosis-inducing ligand |
|  | tumor necrosis factor superfamily member 10 |
|  | TN10 |
|  | TL2 |
|  | Ly81 |
|  | Tnfsf10 |
|  | A330042I21Rik |
|  | A330042I21 |
|  | TN10\_HUMAN |
|  | GNB:1149558 |
|  | tumor necrosis factor (ligand) family, member 10 |
|  | trail/apo2l |
|  | CD253 antigen |
|  | TNF superfamily member 10 |
|  | Tnlg6a |
|  | TRAIL |
|  | TNF-related apoptosis inducing ligand TRAIL |
|  | APO2L |
|  | APO-2Ls |
|  | tumor necrosis factor apoptosis-inducing ligand splice variant delta |
|  | RIKEN cDNA A330042I21 gene |
|  | antigen CD253 |
|  | tumor necrosis factor (ligand) superfamily, member 10 |
|  | CD253 |
|  | AI448571 |
|  | Apo-2 ligand |
|  | tumor necrosis factor ligand 6a |
|  | TNF-related apoptosis inducing ligand |
|  | chemokine tumor necrosis factor ligand superfamily member 10 |
|  | Apo-2L |
|  | tumor necrosis factor ligand superfamily member 10 |
|  | TNF-related apoptosis-inducing ligand |
|  | tumour necrosis factor (ligand) family, member 10 |
|  | tumor necrosis factor (ligand) superfamily member 10 |
|  | tumour necrosis factor apoptosis-inducing ligand splice variant delta |
|  | tumour necrosis factor superfamily member 10 |
|  | tumour necrosis factor-related apoptosis-inducing ligand |
|  | chemokine tumour necrosis factor ligand superfamily member 10 |
|  | tumour necrosis factor (ligand) superfamily, member 10 |

---

|  |  |
| --- | --- |
| GO ID | 0045569 |
|  | 0005125 |
|  | 0005102 |
|  | 0005164 |
|  | 0008270 |
|  | 0006919 |
|  | 0006915 |
|  | 0007166 |
|  | 0007267 |
|  | 0006955 |
|  | 0008584 |
|  | 1902042 |
|  | 0043123 |
|  | 0043065 |
|  | 0043280 |
|  | 2001238 |
|  | 0090200 |
|  | 1902041 |
|  | 0032868 |
|  | 0007165 |
|  | 0070062 |
|  | 0005576 |
|  | 0005887 |
|  | 0046872 |
|  | 0032813 |
|  | 0005615 |
|  | 0016021 |
|  | 0016020 |
|  | 0005886 |
|  | 0010469 |
|  | 0006917 |
|  | 0008633 |
|  | 0097296 |
|  | 2001239 |
|  | 0008624 |
|  | 0005515 |
|  | 0097190 |

---

|  |  |
| --- | --- |
| KEGG ID | hsa:8743 |
|  | mmu:22035 |
|  | rno:246775 |

---

|  |  |
| --- | --- |
| Organism | Homo sapiens {Organism urn:agi-taxid:9606} |
|  | Mus musculus {Organism urn:agi-taxid:10090} |
|  | Rattus norvegicus {Organism urn:agi-taxid:10116} |
|  | Homo sapiens |
|  | Mus musculus |
|  | Rattus norvegicus |

---

|  |  |
| --- | --- |
| Mouse chromosome position | 3 |

---

|  |  |
| --- | --- |
| OMIM ID | 603598 |

---

|  |  |
| --- | --- |
| Rat chromosome position | 2q24 |

---

|  |  |
| --- | --- |
| Hugo ID | 11925 |
|  | HGNC:11925 |

---

|  |  |
| --- | --- |
| Human chromosome position | 3q26.31 |
|  | 3q26 |

---

|  |  |
| --- | --- |
| Swiss-Prot Accession | P50591 |
|  | Q6IBA9 |
|  | P50591.1 |
|  | P50592 |
|  | Q3U5H0 |
|  | P50592.1 |
|  | E9PU84 |
|  | A1Y9B3 |
|  | Q8K3G0 |

---

|  |  |
| --- | --- |
| GenBank ID | NC\_000003 |
|  | NR\_033994 |
|  | NM\_001190942 |
|  | NP\_001177871 |
|  | NM\_003810 |
|  | NP\_003801 |
|  | NM\_001190943 |
|  | NP\_001177872 |
|  | AC007919 |
|  | AC016938 |
|  | AF178756 |
|  | CH471052 |
|  | EAW78465 |
|  | EAW78466 |
|  | EAW78467 |
|  | CQ892411 |
|  | CAH68809 |
|  | CS276168 |
|  | CAL33971 |
|  | EF064708 |
|  | ABK41891 |
|  | HA637646 |
|  | CAZ39545 |
|  | JA362607 |
|  | CCA94584 |
|  | KT258720 |
|  | AK296085 |
|  | BAG58840 |
|  | AK309200 |
|  | AK312742 |
|  | BAG35612 |
|  | AY312579 |
|  | AAR16184 |
|  | BC009795 |
|  | BC020220 |
|  | BC032722 |
|  | AAH32722 |
|  | BT019564 |
|  | AAV38371 |
|  | CR456895 |
|  | CAG33176 |
|  | DA041872 |
|  | DA969653 |
|  | DQ848564 |
|  | ABI24016 |
|  | EU183231 |
|  | ABW24658 |
|  | LN874323 |
|  | CTQ86088 |
|  | U37518 |
|  | AAC50332 |
|  | U57059 |
|  | AAB01233 |
|  | P50591 |
|  | NC\_000069 |
|  | NM\_009425 |
|  | NP\_033451 |
|  | AB052771 |
|  | BAB19680 |
|  | AC116585 |
|  | AC157919 |
|  | AF470537 |
|  | AAM91908 |
|  | CH466530 |
|  | EDL34926 |
|  | AK039433 |
|  | AK142960 |
|  | BAE25231 |
|  | AK144931 |
|  | AK153581 |
|  | BAE32108 |
|  | AK157633 |
|  | BAE34141 |
|  | BC011469 |
|  | BC138807 |
|  | AAI38808 |
|  | BC138808 |
|  | AAI38809 |
|  | BY734631 |
|  | LN874399 |
|  | CTQ86164 |
|  | U37522 |
|  | AAC52345 |
|  | P50592 |
|  | NC\_005101 |
|  | XM\_017590632 |
|  | XP\_017446121 |
|  | XM\_017590634 |
|  | XP\_017446123 |
|  | XM\_017590633 |
|  | XP\_017446122 |
|  | NM\_145681 |
|  | NP\_663714 |
|  | AC\_000070 |
|  | AABR07009804 |
|  | AABR07009805 |
|  | AAHX01015916 |
|  | AAHX01015917 |
|  | AAHX01015918 |
|  | CH473961 |
|  | EDM01114 |
|  | AY115578 |
|  | AAM49797 |
|  | EF030546 |
|  | ABK32522 |
|  | FQ225256 |
|  | FQ234188 |
|  | LN874413 |
|  | CTQ86178 |
|  | ABM81799 |
|  | AAHY01024976 |
|  | AAHY01024975 |
|  | DQ890873 |
|  | ABM84955 |
|  | DQ894029 |
|  | AC\_000025 |
|  | AABR06015456 |
|  | AMYH02008147 |
|  | AABR06015457 |
|  | AABR06015458 |
|  | ABBA01058648 |
|  | AC\_000135 |
|  | NC\_018914 |

---

|  |  |
| --- | --- |
| Swiss-Prot ID | TNF10\_HUMAN |
|  | TNF10\_MOUSE |

---

|  |  |
| --- | --- |
| Cell Localization | Cell membrane |
|  | Secreted |
|  | Membrane |
|  | Extracellular |

---

|  |  |
| --- | --- |
| Ensembl ID | ENSG00000121858 |
|  | ENSP00000389931.2 |
|  | ENST00000420541.6 |
|  | ENSP00000241261.2 |
|  | ENST00000241261.7 |
|  | ENSMUSG00000039304 |
|  | ENSMUSP00000040271.5 |
|  | ENSMUST00000046383.11 |
|  | ENSRNOG00000013269 |
|  | ENSRNOP00000017758.5 |
|  | ENSRNOT00000017758.5 |
|  | ENST00000241261 |
|  | ENSP00000389931 |
|  | ENST00000420541 |
|  | ENSRNOP00000017758 |
|  | ENSRNOT00000017758 |
|  | ENSP00000241261 |
|  | ENST00000241261.6 |
|  | ENSMUSP00000040271 |
|  | ENSMUST00000046383 |

---

|  |  |
| --- | --- |
| MGI ID | MGI:107414 |
|  | 107414 |

---

|  |  |
| --- | --- |
| RGD ID | 628734 |

---

|  |  |
| --- | --- |
| Unigene ID | Mm.1062 |
|  | Rn.83627 |
|  | Rn.133346 |
|  | Hs.478275 |

---

|  |  |
| --- | --- |
| Homologene ID | 2824 |

---

|  |  |
| --- | --- |
| Shape | Rhomb |

---

|  |  |
| --- | --- |
| IPI ID | IPI00000049 |
|  | IPI00119330 |
|  | IPI00790933 |

---
